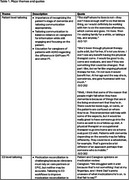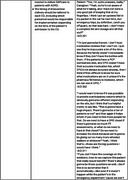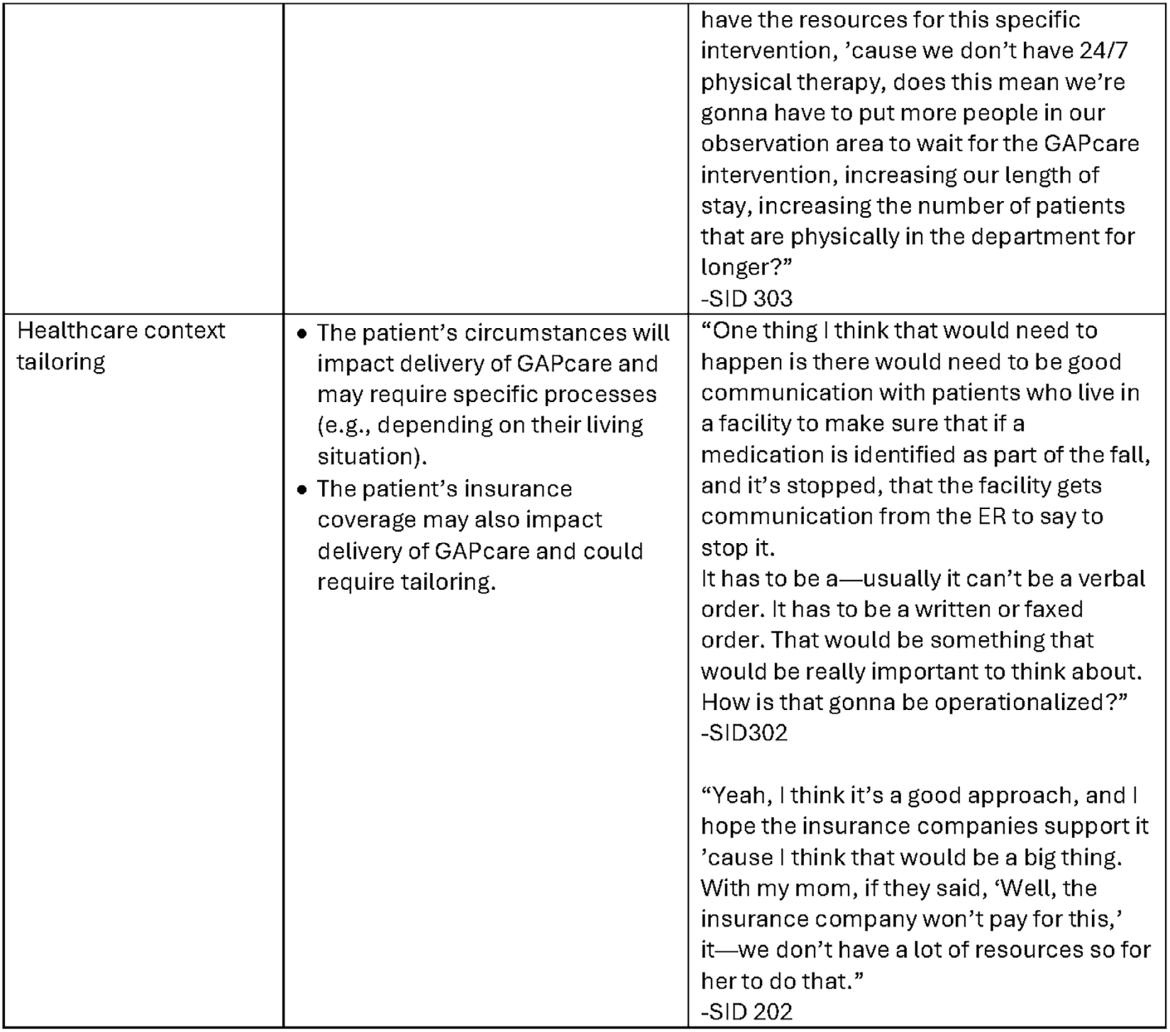# Modifying the GAPcare Fall Prevention Intervention with Patients with Cognitive Impairment and Their Caregivers

**DOI:** 10.1002/alz70858_100546

**Published:** 2025-12-24

**Authors:** Elizabeth M Goldberg, Megan Bounds

**Affiliations:** ^1^ University of Colorado School of Medicine, Aurora, CO, USA; ^2^ University of Colorado Anschutz Medical Campus School of Medicine, Aurora, CO, USA

## Abstract

Although falls are up to eight times more common in persons living with dementia (PLWD), limited fall prevention interventions exist for this population. Adapting promising fall prevention interventions for unique needs of PLWD, such as the GAPcare intervention, which reduced fall related emergency department (ED) visits by 66% and did not prolong ED length of stay, may address this need. In GAPcare, patients receive pharmacy and physical therapy (PT) consultation while in the ED to reduce modifiable risk factors for falls prior to ED discharge.

We conducted semi‐structured interviews with PLWD who recently visited the ED (*n* = 7), their caregivers (*n* = 2), and national experts in dementia care or ED operations (*n* = 15: 5 physicians, 3 nurses, 3 PTs, 3 pharmacists, 1 PhD scientist) to elicit perspectives on how GAPcare should be adapted for PLWD. We conducted Interviews in English and Spanish. We analyzed interviews using rapid qualitative analysis guided by Castro's framework for adapting interventions.

Participants strongly supported improved ED falls care for PLWD. They also indicated that tailoring at multiple levels (patient and caregiver, ED, and external factors, e.g., insurance status) would be required to support the complexities in PLWDs’ circumstances (e.g., living arrangements, income) and cognitive abilities. Contextual aspects of an ED (urgency of visits and pressures to limit length of stay) make it challenging to implement a more complex, comprehensive intervention for PLWD who may have communication difficulties. We recognize the need to simplify the intervention and provide more time for education, explanations and reinforcement, and coordination of care. Balancing the suggestions to enrich the intervention, while also being mindful of ED staff burden and efficiency needs, will be an important challenge to overcome during implementation.

PLWD, caregivers, and experts in dementia care and ED operations are supportive of adapting our existing GAPcare intervention for PLWD. Early feedback from relevant informants guided GAPcareAD intervention adaptation and fit within ED workflows. Lessons learned in enhancing recruitment and retention for this study included offering dyadic and individual interviews for PLWD/caregivers, providing options for in‐person and virtual interviews, and keeping the inclusion criteria broad to improve representativeness.